# An Analog Gamma Correction Scheme for High Dynamic Range CMOS Logarithmic Image Sensors

**DOI:** 10.3390/s141224132

**Published:** 2014-12-15

**Authors:** Yuan Cao, Xiaofang Pan, Xiaojin Zhao, Huisi Wu

**Affiliations:** 1 College of Electronic Science and Technology, Shenzhen University, Shenzhen 518060, China; E-Mail: caoyuan0908@gmail.com; 2 Department of Electronic and Computer Engineering, the Hong Kong University of Science and Technology, Clear Water Bay, Hong Kong, China; E-Mail: xpan@ust.hk; 3 College of Computer Science and Software Engineering, Shenzhen University, Shenzhen 518060, China; E-Mail: hswu@szu.edu.cn

**Keywords:** CMOS image sensor, analog gamma correction, high dynamic range, VCO-based ADC

## Abstract

In this paper, a novel analog gamma correction scheme with a logarithmic image sensor dedicated to minimize the quantization noise of the high dynamic applications is presented. The proposed implementation exploits a non-linear voltage-controlled-oscillator (VCO) based analog-to-digital converter (ADC) to perform the gamma correction during the analog-to-digital conversion. As a result, the quantization noise does not increase while the same high dynamic range of logarithmic image sensor is preserved. Moreover, by combining the gamma correction with the analog-to-digital conversion, the silicon area and overall power consumption can be greatly reduced. The proposed gamma correction scheme is validated by the reported simulation results and the experimental results measured for our designed test structure, which is fabricated with 0.35 μm standard complementary-metal-oxide-semiconductor (CMOS) process.

## 1. Introduction

The past few years have witnessed the rapid development of complementary-metal-oxide-semiconductor (CMOS) image sensors and their wide range of applications in consumer electronics, such as digital cameras, camcorders, smart mobile phones, to name but a few [1]. In various imaging applications, including automobile, aerospace imaging and security, CMOS image sensors capable of sensing the light illumination with high dynamic range (DR) are demanded in order to reconstruct an image covering a wide illumination range (e.g., >100 dB) [2–4]. The conventional image sensor responding linearly to the input light intensity can only have a dynamic range of 60–70 dB. To expand the dynamic range, the direct current conversion mode logarithmic sensor based on the subthreshold operation of MOSFETs has been developed [5–7]. The direct current conversion mode logarithmic sensor features a current mirror configuration and a log sensor structure. When the photocurrent is so small, the transistor enters the subthreshold region. However, the wide DR of image sensor exerts stringent requirements on many image signal processing techniques to enhance the image quality [8], among which, the most representative one is gamma correction [9]. Gamma correction is a mainstream technique to improve image quality [10]. It is used to compensate and correct the errors caused by the non-linear response of modern CMOS image sensors. The non-linear luminance produced by these image sensing devices can be described by the following power-law expression [10]:

(1) $$f\left(x\right)=x^{\frac{1}{\gamma }}$$

where *x* represents the input voltage, which is often normalized between 0 and 1. And γ is a coefficient with its value determined by experiments.

**Figure 1.**
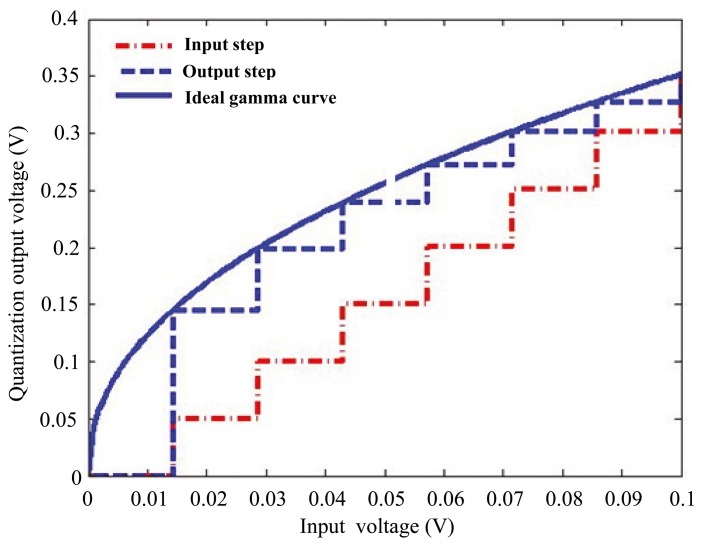
Illustration of gamma correction's quantization effects in low luminance regions.

Gamma correction is traditionally conducted in digital domain. Direct lookup tables are typically exploited to realize the digital gamma correction [11]. However, the resolution and dynamic range of the analog-to-digital converter (ADC) are always limited, which largely degrades the image quality, especially with low light illumination. Specifically, in the low light region, the slope of the gamma correction curve is larger than 1, leading to an amplification of the ADC's quantization error [12]. Moreover, as shown in Figure 1, when the input's quantization step is smaller than that of the output, the system is disabled to generate the low luminance levels at the output [12]. Although this issue can be addressed with higher resolution ADC, it can result in an exponential increase of storage amount dedicated to the direct lookup tables. Furthermore, for the high dynamic range applications, the requirements on input resolution and memory resources become much more stringent [12]. Based on non-linear single slope ADC, two analog gamma correction schemes have been reported to avoid the amplification of the quantization error [13,14]. Nevertheless, in [13], the whole dynamic range is not fully utilized. The implementation reported in [14] suffers from the design complexity of a non-linear ramp generator. What is more, in [14], as the gamma value is not constant in the whole dynamic range, it is quite challenging to extend the reported structure to high dynamic range applications.

In this paper, we propose a novel gamma correction scheme with a voltage-controlled-oscillator (VCO)-based ADC. The proposed VCO-based ADC features a non-linear output approximate to typical gamma correction curve, which fully avoids the adopted ramp generator and the quantization error amplification in the previous implementations. Additionally, the analog to digital conversion process is included in our proposed gamma correction scheme, leading to significant silicon area and power consumption saving of the on-chip very-large-scale-integration (VLSI) implementation. Moreover, the proposed gamma scheme is further applied to logarithmic image sensors to achieve high dynamic range and its effectiveness is validated by the reported simulation and experimental results. The remaining of the paper is organized as follows: Section 2 presents the proposed logarithmic image sensor architecture with on-chip correlated double sampling (CDS). Section 3 provides the VLSI implementation of the VCO-based ADC. The proposed circuitry is validated by the reported simulation and experimental results in Section 4. Finally, this paper is concluded in Section 5.

## 2. Logarithmic Image Sensor with On-Chip CDS

**Figure 2.**
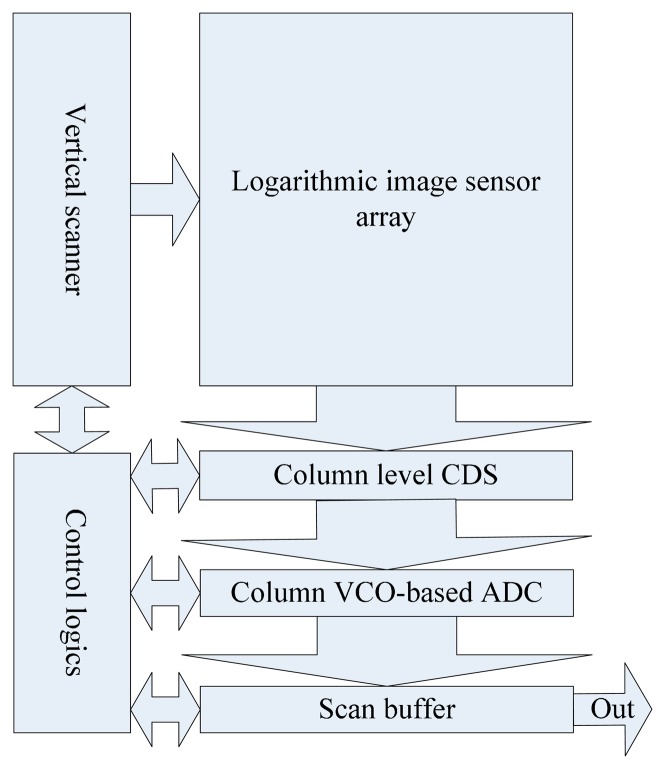
The proposed sensor architecture including a logarithmic image sensor with on-chip CDS and a VCO based ADC.

Figure 2 presents the proposed sensor architecture, which is composed of a logarithmic image sensor with on-chip CDS mechanism and a novel VCO-based ADC. It is known that the CMOS logarithmic image sensor features high dynamic range of the illumination light [15]. However, it is quite sensitive to fixed pattern noise (FPN), which is mainly introduced during fabrication due to the pixel-to-pixel parameter variation. In [15], the FPN was modeled by the response of each pixel in terms of offset voltage, gain and leakage current. Based on this, a number of techniques have been proposed to suppress the FPN [15,16]. These techniques share the same principle, that is, the individual pixel offsets can be corrected by extracting the difference between the output signals before and after the integration. However, for the pixel of the logarithmic image sensor, the conversion of light intensity to voltage is continuous. Therefore, a different scheme is needed to remove the offset voltage. In this work, we adopt an in-pixel CDS technique in order to alleviate the influence of FPN. Different from the previously reported on-chip calibration method [17], a current source is employed instead of the leakage current of a transistor for this calibration in order to increase the system's overall speed.

**Figure 3.**
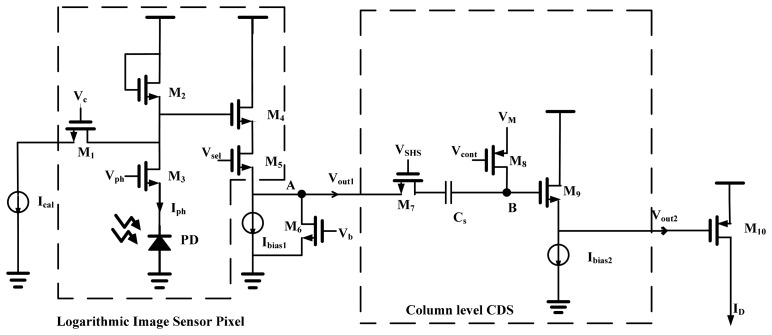
The schematic of logarithmic image sensor pixel with on-chip CDS.

As shown in Figure 3, in the logarithmic image sensor's pixel, we connect the control signals *V_c_* and *V_b_* (denoted as *V_Body_*) of each pixel in the same column of the logarithmic image sensor's pixel array, and there are only two current sources *I_cal_* and *I_bias_*_1_ in each column. This column-parallel processing can greatly improve the image sensor's processing speed. In addition, this logarithmic sensor is able to provide two voltage output levels for removing the offset by the following CDS circuit. At the beginning, *V_c_* is set to low and *V_ph_* is set to high in the signal readout phase. The pixel output *V_sig_* at node *A* is expressed as follows [18]:

(2) $$V_{\text{sig}}=G_{1}\left(V_{dd}-V_{th:M_{2}}-nV_{t}\ln\left(\frac{I_{\text{sig}}}{I_{0}}\right)\right)-V_{th:M_{4}}$$

where *V_dd_* is the power supply voltage, *V_t_* is the thermal voltage and *G*_1_ is the gain of the source follower *M*_4_. *V_th:M_*_2_ and *V_th:M_*_4_ are the threshold voltages of the bias transistor *M*_2_ and the transistor *M*_4_, respectively. *I*_0_ is the drain current with *V_GS:M_*_2_ = 0, and *n* is the subthreshold slope factor usually approximate to 1, both of which are process dependent parameters. *I_sig_* is the photocurrent passing the load transistor *M*_2_. In Equation (2), the pixel-to-pixel threshold voltage variation of *M*_2_ and *M_4_* (*i.e.*, *V_th:M_*_2_ and *V_th:M_*_4_) can introduce non-neglectable change of *V_sig_*. This variation is dependent on the manufacturing process and corresponds to a non-ideal effect known as fixed pattern noise (FPN) [6]. As a result, even under the uniform illumination, the voltage output of each pixel is slightly different from the other pixels. During the calibration phase, *V_c_* is set to high and *V_ph_* is set to low. According to Equation (2), the new output *V_cal_* of node *A* is calculated by replacing *I_sig_* with *I_cal_*. Therefore *V_cal_* is expressed as:

(3) $$V_{\text{cal}}=G_{1}\left(V_{dd}-V_{th:M_{2}}-nV_{t}\ln\left(\frac{I_{\text{cal}}}{I_{0}}\right)\right)-V_{th:M_{4}}$$

In order to keep *M*_1_ operate in the subthreshold region with a short settling time, the calibration current *I_cal_* is maintained at an appropriate range.

Moreover, the source follower *M*_4_ is implemented with its gain tunable. According to [15], the gain of the source follower *G*_1_ is equal to:

(4) $$G_{1}=\frac{g_{m}R_{s}}{1+g_{m}R_{s}}$$

where *R_S_* is the resistance of the load and *g_m_* is the transconductance. It is indicated by Equation (4) that the source follower's gain is controlled by *R_S_*, and the load resistance *R_S_* is adjusted by the added transistor *M*_6_ working in the linear region.

**Figure 4.**
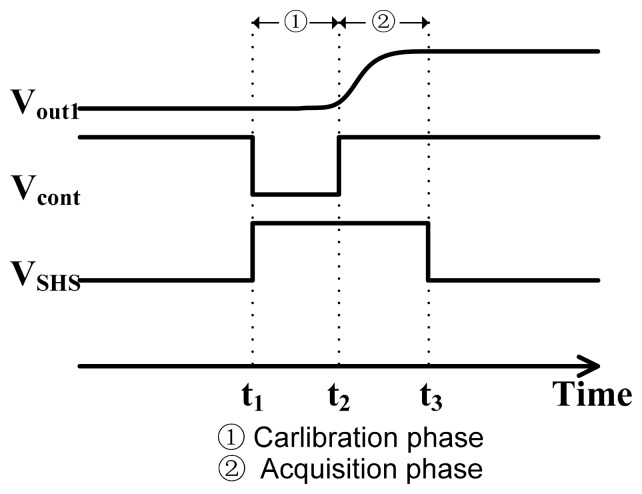
The timing diagram of the CDS operation.

Here CDS technique is exploited to eliminate the FPN by measuring the output of the sensor twice: the first one in reset phase and the second one when the charges are transferred to the read-out node. Successively, these two signals are differentiated at the following stage. Figure 4 illustrates the timing diagram of the CDS. First, node *B* is connected to an external bias *V_M_* (1.5 V) via *M*_8_ at *t*_1_, and then the calibration voltage *V_cal_* is readout before *t*_2_. The sampling capacitor *C_S_* stores the voltage of *V_cal_* + *V_pix_offset_* − *V_M_*, where *V_pix_offset_* is the total equivalent pixel voltage offset. After node *B* is floating by turning off *M*_8_ at *t*_2_, the pixel signal value *V_sig_* + *V_pix_offset_* is then readout at *t*_3_, which makes node *B* voltage *V_B_* equivalent to:

(5) $$V_{B}=V_{\text{sig}}-V_{\text{cal}}+V_{M}$$

According to Equation (2), the two offset variables *V_th:M_*_2_ and *V_th:M_*_4_ can be eliminated by the CDS circuit, due to the final output *V_B_* of CDS expressed as:

(6) $$V_{B}=G_{1}nV_{t}\ln\left(\frac{I_{\text{cal}}}{I_{\text{sig}}}\right)+V_{\text{M}}$$

As a result, the CDS output *V_out_*_2_ is equal to:

(7) $$V_{\text{out}2}=G_{2}V_{B}-V_{th:M_{9}}$$

where *G*_2_ is the gain of the source follower in the CDS, and *V_th:M_*_9_ is the threshold voltage of *M*_9_. Therefore, according to Equation (6), *V_out_*_2_ can be rewritten as:

(8) $$V_{\text{out}2}=G_{2}\;\left(G_{1}nV_{t}\ln\left(\frac{I_{\text{cal}}}{I_{\text{sig}}}\right)+V_{\text{M}}\right)-V_{th:M_{9}}$$

Through the technique of CDS, both the variations of threshold voltage and *I*_0_ in the pixel array are eliminated, which correspond to the most important FPN sources. Then the output voltage *V_out_*_2_ can be used to control the drain current of the following bias transistor *M*_10_ for the VCO-based ADC.

## 3. VLSI Implementation of the Proposed VCO-Based ADC

In this section, a novel implementation of VCO-based ADC is presented. In each sampling period, a frequency counter is utilized to calculate the number of generated pulses at the VCO's output node. The value stored in the counter just represents the quantized estimation of the analog input signal.

**Figure 5.**
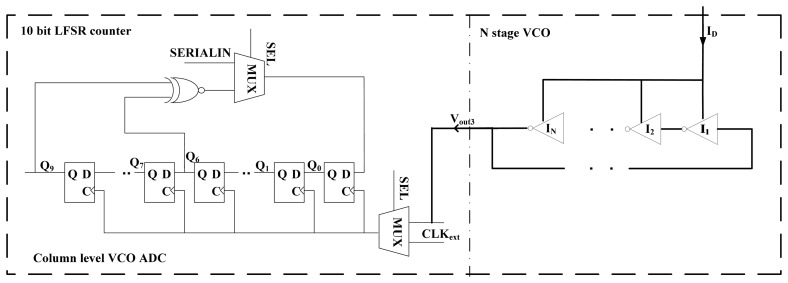
The schematic of the proposed VCO-based ADC.

As illustrated in Figure 5, the proposed VCO consists of an odd number (*N*) of inverters connected in a loop. Suppose the NMOS and PMOS transistors have equal driving ability and the delay time of each inverter is *t_d_*, the formed oscillator's output frequency is:

(9) $$f=\frac{1}{2Nt_{d}}$$

Assume the capacitance load of each node in the inverter chain is *C_L_*, the delay in the inverter exists due to the time needed for the transistors in the inverter to charge *C_L_*. If *N* is large enough, all nodes will be completely charged and discharged during one period, and each inverter delivers the charge of *C_L_* × *V_dd_*. As a result, the capacitance *C_L_* is initially charged with a maximum current *I_D_*, and the current decreases during the transition. Given that η*I_D_* is the average current (disregarding leakage current), *t_d_* can be formulated as:

(10) $$t_{d}=\frac{C_{L}V_{dd}}{\eta I_{D}}$$

Therefore, the frequency of the VCO is expressed as:

(11) $$f=\frac{\eta I_{D}}{2NC_{L}V_{dd}}$$

where *N* and η are fixed parameters for a given VCO. The power supply of *M*_10_ is separated from the other modules in the sensor, which is set to be 0.7 V externally to make the bias transistor *M*_10_ always work in the subthreshold region. Therefore, we can derive the drain current *I_D_* of *M*_10_:

(12) $$I_{D}=I_{1}\exp\left(\frac{-\left|V_{GS:M_{10}}-V_{th:M_{10}}\right|}{nV_{t}}\right)$$

where *I*_1_ is constant. In this structure, *V_GS:M_*_10_ = *V_out_*_2_ − *V_dd_*. By combining Equations (8) and (12), we can have:

(13) $$I_{D}=I_{1}\exp\left(\frac{{\text{V}}_{\text{th}:{\text{M}}_{9}}+{\text{V}}_{\text{dd}}+{\text{V}}_{\text{th}:{\text{M}}_{10}}-{\text{G}}_{2}V_{\text{M}}}{nV_{t}}\right)*{\left(\frac{I_{\text{sig}}}{I_{\text{cal}}}\right)}^{{\text{G}}_{1}{\text{G}}_{2}}=K*{\left(\frac{I_{\text{sig}}}{I_{\text{cal}}}\right)}^{{\text{G}}_{1}{\text{G}}_{2}}$$

where *K* is a constant if *G*_1_, *G*_2_ and *V_M_* are fixed. Since the output frequency *f* is linearly proportional to the *I_D_*, we can have:

(14) $$f=\alpha *{\left({\text{I}}_{\text{sig}}\right)}^{{\text{G}}_{1}{\text{G}}_{2}}$$

where α is constant. It is indicated that the digitized readout for a pixel has an exponential relationship with the input light intensity and the power can be modified by *G*_1_ and *G*_2_.

In addition, as shown in Figure 5, a 10-bit linear feedback shift register (LFSR) counter with dynamic D flip-flops is adopted in this design [19]. The input signal *SEL* is utilized for mode switching. If *SEL* is high, the counter is in the counting mode. If *SEL* is low, the pixel is in the data readout mode. *CLK_ext_* is the clock signal in the data readout mode, and *SERIALIN* is the data input port to reset the counter. It is noted that the output of the VCO cannot achieve full swing and a voltage level shifter is required. Here we embed a fast and low power consumption voltage level shifter to the MUX for the clock input of the counter [20]. In order to remove the column level FPN, we firstly measured the data D in LFSR when there is no light illuminated on the sensor. The data D represents the column level FPN. We initialized the LFSR with the complementary value of data D at the beginning of every AD conversion when SEL for the MUX is set to 0. After that, the LFSR starts to count the pixel output with light illuminated on the sensor when SEL is set to 1. Through this way, the column level FPN can be reduced. The advantages of using the LFSR counter include: (1) the counter design is simple with a regular and compact layout; (2) the complexity of the counter does not increase with the counter length.

Furthermore, the proposed nonlinear ADC corresponds to an analog gamma correction, which features higher signal-to-noise ratio (SNR) than the digital gamma correction, especially for the low signal level. Suppose the input signal *S* of the ADC is:

(15) $$S=P+n_{c}$$

where *P* is the pixel signal, and *n_c_* is the circuit noise. The output of the linear ADC can be expressed as:

(16) $$D_{\text{out}}=P+n_{c}+n_{dq}$$

where *D_out_* is the output of linear ADC and *n_dq_* is the quantization noise for the digital gamma correction method. As a result, the output of the digital gamma correction *G_d_* can be expressed as:

(17) $$G_{d}=A*D_{\text{out}}+n_{t}=A*\left(\text{P}+n_{\text{c}}\right)+A*{\text{n}}_{\text{dq}}+n_{\text{t}}$$

where *A* is the gamma correction curve and *n_t_* is the truncation error in digital gamma correction; while the output *G_a_* of the proposed VCO based ADC is:

(18) $$G_{a}=A*S+n_{aq}=A*\left(\text{P}+n_{c}\right)+n_{aq}$$

where *n_aq_* is the quantization noise for the analog gamma correction method. If the digital gamma correction uses the same ADC architecture, *n_aq_* should be the same as *n_dq_*. From Equations (17) and (18), we can see that the analog gamma correction has a constant quantization noise over the entire signal range while the digital gamma correction has amplified quantization noise at the low signal level (*A* > *1* for the low signal level).

## 4. Simulation and Experimental Results

**Figure 6.**
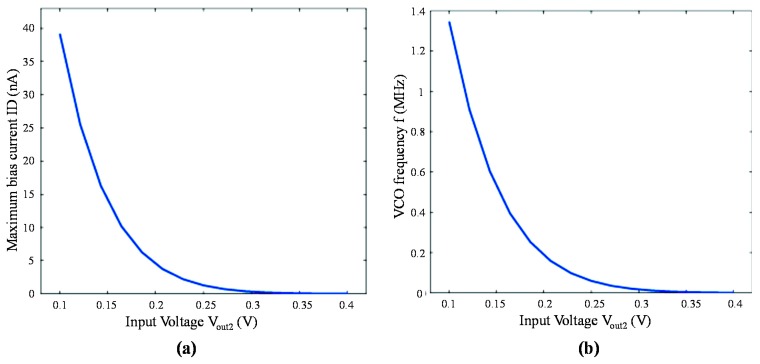
The simulation results of (**a**) the maximum bias current *I_D_* and (**b**) the VCO frequency as functions of bias voltage *V_out_*_2_.

In this section, RF simulations with the *Spectre* tool of Cadence were conducted based on the standard 0.35 μm CMOS process. Two most important figures of merit (*i.e.*, maximum current *I_D_* of the bias transistor *M*_10_ and the VCO output frequency *f*) with different bias voltages are calculated and plotted Figure 6a,b, respectively. The curve in Figure 6 shows excellent agreement with Equation (13). In addition, it is indicated that the frequency output of the VCO has the same logarithmic relationship with the maximum current, which is in accordance with Equation (14).

In the simulation, the *V_DS_* over the bias transistor *M*_10_ is also taken into account, as a result, the amplitude of the VCO output cannot achieve the full swing. On the other hand, the soft rail negative feedback exists as the change of *V_DS_*, which has no influence on the output frequency signal. Here we propose a more accurate model for the drain current of the bias transistor *M*_10_ to explain this effect, where *I_D_* is expressed as:

(19) $$I_{D}=I_{1}\exp\left(\frac{-\left|V_{GS:M_{10}}-V_{th:M_{10}}\right|}{nV_{t}}\right)\left(1-e^{-\delta \frac{V_{DS}}{V_{t}}}\right)$$

It is indicated by Equation (19) that *I_D_* increases as *V_DS_* increases. In contrast, the increase of *V_DS_* leads to the decrease of the supply voltage for the inverters of VCO, which reduces the *I_D_*. With proper sizing of the bias transistor *M*_10_, the soft rail negative feedback can alleviate the body effect caused by *V_BS_*.

**Figure 7.**
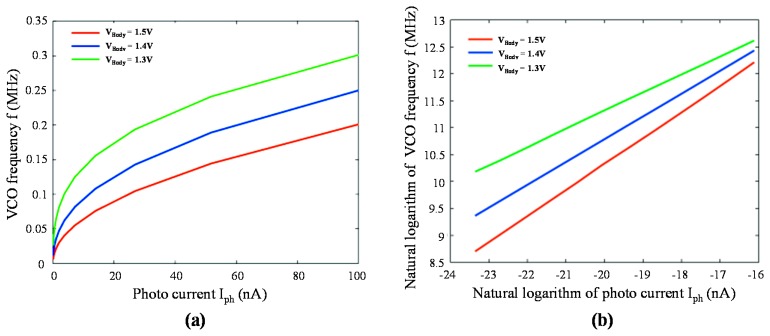
The simulated VCO frequency *vs.* photocurrent with different *V_Body_* in (**a**) linear space; (**b**) natural logarithmic space.

Figure 7a,b present the simulation results of the proposed structure's frequency response *f* as a function of photocurrent *I_ph_* with variable *V_Body_* (*i.e.*, *V_b_*) in linear space and logarithmic space, respectively. According to Equation (14), after natural logarithm performed on both sides of the equation, we can have:

(20) $$\ln\left(f\right)={\text{G}}_{1}{\text{G}}_{2}*\ln\left({\text{I}}_{ph}\right)+\ln\left(\text{C}\right)$$

From Equations (1) to (20), we notice that the slope *G*_1_*G*_2_ is equal to 1/γ, which means the γ value can be adjusted by *G*_1_ and *V_Body_* (*G*_2_ is fixed, not tunable). The simulation results of the relation between *V_Body_* and the slope (1/γ) are tabulated in Table 1.

**Figure 8.**
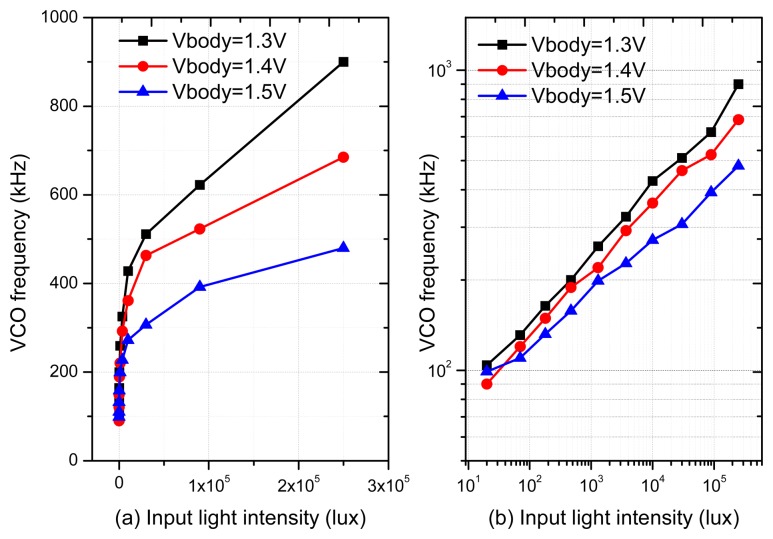
The measured VCO frequency from the test pixel structure with input light intensity ranging from 0 to 250 klux: (**a**) linear space; (**b**) logarithmic space.

In order to study the relationship between the VCO frequency and the input light intensity, a single pixel with the VCO-based ADC has been fabricated using 0.35 μm standard CMOS process. The VCO consists of 25 inverter stages. The digital 1/1024 frequency divider has been employed to slow down the frequency, as the high frequency signal is extremely hard to be output through the normal I/O pad. With a model 66885 light source from Newport Corp. (Irvine, CA, USA), Figure 8a,b show the measured VCO frequency *vs.* light intensity for a test pixel in linear space and logarithmic space, respectively. We can see the proposed method can provide an adjustable gamma value over a wide dynamic range. Typically, the measured curve slope of 0.47 corresponds to a gamma value of 2.13 when *V_Body_* = 1.4 V.

**Figure 9.**
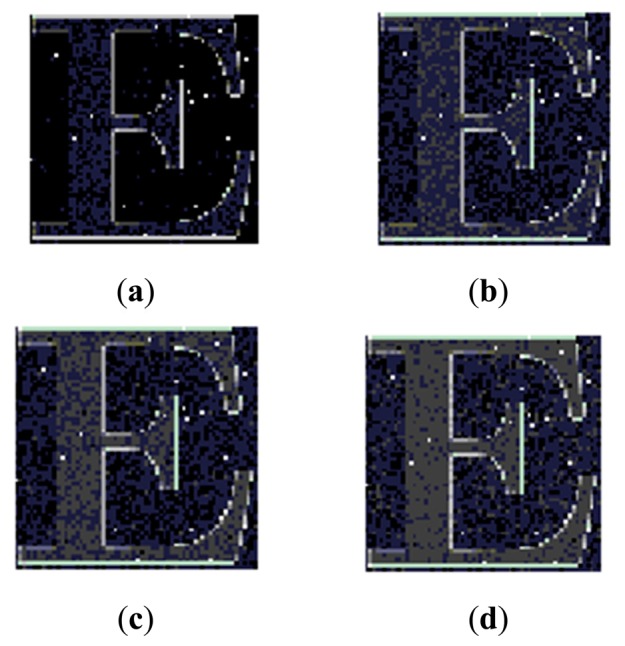
Photos taken by the fabricated 64 × 64 test image sensor with different body bias (*V_Body_*). (**a**) *V_Body_* = 1.1 V; (**b**) *V_Body_* = 1.4 V; (**c**) *V_Body_* = 1.5 V; (**d**) *V_Body_* = 1.6 V.

Furthermore, the proposed image sensor structure with a resolution of 64 × 64 was fabricated with the 0.35 μm standard CMOS process. Figure 9 shows the pictures taken by the fabricated 64 × 64 test image sensor with different body bias (*V_Body_*). The contrast of the image can be enhanced with a different gamma value by adjusting *V_Body_*. Finally, the test structure's main characteristics and the detailed comparison with the previous analog gamma correction methods are summarized in Table 2. In this table, the dynamic range (DR) is calculated by [21]:

(21) $$DR=20\log\left(\frac{lux_{\text{max}}}{lux_{\text{min}}}\right)$$

where *lux_max_* and *lux_min_* are the maximum and the minimum light intensity which can be sensed. In [3], a CMOS image sensor with tunable dynamic range has been proposed. The logarithmic curve in the pixel is used to approximate the gamma curve, which can reach a very high DR (e.g., 112 dB). Moreover, a frequency modulation counter that can realize the anolog gamma correction has been proposed and implemented in [22]. In order to obtain a higher ADC gain in the dark light levels and a lower ADC gain in the bright light levels, the counter reduces the counting number of the pulses through a constant time interval. In contrast to the other analog gamma correction methods, the proposed method can reach a high DR of 82 dB without complex ramp generator. The measured noise floor in our implementation is 58 dBuV. In addition, the proposed method has integrated the gamma correction into the A/D conversion. Hence, the total power consumption and the area per pixel can be significantly reduced as shown in the Table 2.

## 5. Conclusions

In this paper, we report an analog gamma correction scheme for logarithmic CMOS image sensor featuring high dynamic range. Compared with the previous implementations, the proposed analog gamma correction scheme exerts no amplification of the quantization errors. In addition, high illumination dynamic range can be achieved with the proposed logarithmic image sensor with on-chip CDS. Moreover, the proposed VCO-based ADC, featuring non-linear output approximate to typical gamma correction curve, is combined in our proposed gamma correction scheme, which leads to significant silicon area and power consumption saving of the VLSI implementation. Furthermore, the proposed gamma correction scheme is validated by the reported simulation and experimental results, which can find CMOS image sensors applications requiring low power, low cost and high dynamic range.

## Figures and Tables

**Table 1.** Simulation results of *V_Body_*, slope and norm of residuals.

***V_Body_* (V)**	**Slope**	**γ**	**Norm of Residuals**
1.30	0.48	2.07	2.17E–2
1.40	0.42	2.36	3.31E–3
1.50	0.33	2.96	2.10E–2

**Table 2.** Main chip characteristics and the detailed comparison with the previous analog gamma correction methods.

	**[14]**	**[3]**	**[22]**	**This Work**
Technology	0.35 μm	0.35 μm	0.13 μm	0.35 μm
Dynamic range (DR)	64.8 dB	112 dB	80 dB	82 dB
Pixel size (μm^2^)	5.6 × 5.6	9.4 × 9.4	2.25 × 2.25	8.5 × 8.5
Fill factor (%)	Not reported	Not reported	30%	42%
Non-linear ramp generator	Needed	No need	Needed	No need
Power dissipation (mW)	30	118	30	10
Core chip size (mm^2^)	Not reported	6.36 × 3.68	1.5 × 2.0	1.28 × 0.93
Pixel array	320 × 240	100 × 100	320 × 240	64 × 64
Average area per pixel (μm^2^)	Not reported	2340	39	290
Frame rate	15 fps	30 fps	37 fps	78 fps
FPN	NA	0.83%	0.15%	0.92%
ADC resolution	10 bit	12 bit	10 bit	10 bit
